# Effects of BDNF Signaling on Anxiety-Related Behavior and Spatial Memory of Adolescent Rats in Different Length of Maternal Separation

**DOI:** 10.3389/fpsyt.2020.00709

**Published:** 2020-07-23

**Authors:** Xianqiang Zhang, Haonan Li, Haoran Sun, Yinghong Jiang, Aihong Wang, Yujia Kong, Xiue Sun, Guohui Zhu, Qi Li, Zhongde Du, Hongwei Sun, Lin Sun

**Affiliations:** ^1^ Department of Psychology, Weifang Medical University, Weifang, China; ^2^ Department of Clinical Medicine, Weifang Medical University, Weifang, China; ^3^ Department of Hematology, Affiliated Hospital of Weifang Medical University, Weifang, China; ^4^ School of Public Health and Management, Weifang Medical University, Weifang, China; ^5^ Department of Nursing, Affiliated Hospital of Weifang Medical University, Weifang, China; ^6^ Depression Treatment Center, Weifang Mental Health Center, Weifang, China; ^7^ Department of Psychiatry and Centre for Reproduction Growth and Development, University of Hong Kong, Hong Kong, Hong Kong; ^8^ Department of Neurology, Sunshine Union Hospital, Weifang, China

**Keywords:** maternal separation, anxiety-related behavior, spatial memory, brain-derived neurotrophic factor, prefrontal cortex

## Abstract

As an adverse form of early-life stress (ELS), maternal separation (MS) can interfere with the development of cognition and behaviors of adolescent rodents. Brain-derived neurotrophic factor (BDNF) is involved in the regulation of brain development and function, but the molecular mechanisms by which BDNF regulates brain function and behavior in MS with different stressor strengths remain unclear. This descriptive study characterized the levels of BDNF in the prefrontal cortex (PFC) and plasma corticosterone (CORT) from the offspring of rats exposed to early handling (EH, 15-min separation per day) and prolonged MS (PMS, 180-min separation per day), during postnatal days (PND) 1‑21. The behavioral and biochemical analyses were performed during adolescence (PND 42‑56). PMS resulted in reduced weight and decreased locomotor activity in the open field test and Y-maze task compared to control (CON) group, with EH showing an intermediate phenotype. BDNF protein levels in the PFC were lower in PMS compared to EH and further reduced in CON male rats. Plasma CORT levels were higher in PMS compared to CON with EH again showing intermediate levels. Neither PMS or EH affected spatial learning in the Y-maze task. These findings indicate that longer periods of maternal separation are necessary to increase anxiety-like behavior, elevate CORT levels, and further suppress BDNF levels in the PFC, providing a possible mechanism to explain why more severe forms of ELS lead to more significant psychiatric and medical consequences later in life.

## Introduction

Early-life stress (ELS) is a broad term used to describe various forms of stress experienced in early life, including physical, sexual, and emotional forms of abuse and neglect suffered by developing children (1), which can alter the normal pattern of brain development, resulting in various short- and long-term dysfunctions in cognitive, emotional, and other behavioral processes (2). Adolescence is a life period in which neurodevelopment is particularly susceptible to social influence. It is thought that early disruptions of social interaction in young children may affect brain development and have long-term neurochemical, endocrine, and behavioral effects (3). Clinical research has found an increased risk for developing depression, post-traumatic stress disorder, and schizophrenia among victims of childhood abuse and neglect (4). More than 50% of children worldwide are estimated to have been exposed to early stress (5), and these children are more likely to suffer from psychological health problem, especially with severe cognitive impairment (6), within a few months or a few years in the absence of a good environment or family support.

Maternal separation (MS) is a rodent model of early adversity in which pups are repeatedly separated from the mother for different periods of times (7, 8). Current evidence indicates that MS leads to some behavioral and neurochemical disorders, such as emotional behavioral deficits, increased locomotor activity, disturbance in serotonergic and dopaminergic activities, and also lead to persistent changes in brain structural plasticity (9). Other studies have shown that repeated MS leads to memory and learning deficits in experiments using the Morris water maze and object recognition tests (10). However, it has also been reported that MS can improve reverse learning in adolescent rats (8). These seemingly conflicting results may reflect experimental differences in the MS procedure, the developmental age at which the testing was performed, or the cognitive test used (11).

Due to the variability of the MS models, the same anxiety and cognitive behavior changes cannot always be found in the early stressed population. Moreover, some studies have reported that mild separation stimuli may enhance the ability to withstand stress in adulthood. The central argument of such studies was based on the premise that exposure to new stimuli during infancy makes animals more resilient to stress (12). In contrast, Lupien found that depriving pups of prolonged maternal contact during postnatal development did not attenuate the stress-induced response, but rather negatively affected their ability to manage future stressors (13).

Clinical data and meta-analysis have confirmed the deleterious effects of adversity such as physical, sexual, and emotional abuse (14), death or separation of parents (15), out of home care for children on the risk of mental illness (16). Importantly, experiencing more or longer periods of early adversity increased the risk of disease, leading to more serious adverse outcomes. Research has shown that the risk increased by approximately 50% in individuals who have experienced one adversity, and by nearly 300% among individuals with four or more adversities (16, 17).

To elucidate the causal effects of early life experiences, rodents have been widely used as models for studying the disruptions of maternal offspring relationships (18). Previous research used protocols that included two postnatal procedures during the postnatal day (PND) 1‑21, namely early handling (EH), which consisted of daily separations of the offspring from their mother for 3‑15 min, and prolonged MS (PMS), which consisted of daily separation for 180 min (19). EH has been reported to have strong effects on the behavior of young adult rats, including a reduction in fear-related behaviors in spontaneous and conditioned tasks (20). The long-term effects of PMS procedures in early adulthood are the opposite of EH, including the increase of fear avoidance behaviors and the hypothalamic-pituitary-adrenal (HPA) axis responses (18, 20). Although it is recognized that exposure to MS as a stressor can seriously affect the balance of neural structure and function in the brain, the mechanisms underlying stress in early life are still not fully understood.

Brain-derived neurotrophic factor (BDNF), which is one of several endogenous proteins, plays a key role in the survival and development of the brain and peripheral neurons (21). A common genetic variation in the BDNF gene, the Val66Met, is associated with specific mood, cognitive and neuroanatomical alterations in disease (22). Research has suggested that BDNF Val66Met carriers with high levels of childhood abuse had more severe cognitive deficits than normal controls (23). In addition, BDNF can directly influence the HPA axis modulation by altering the expression levels of corticotropin releasing hormone, thereby affecting the evaluation and memory retrieval of novel information in the brain, and regulating the behavioral coping responses (24, 25). Previous studies have demonstrated that single or repeated MS caused a significant reduction in the expression of the neurotrophin BDNF in adulthood (26). However, previous studies did not find significant changes in the MS-induced alterations in BDNF in the prefrontal cortex (PFC) (27). These studies reported inconsistent behavioral and neurochemical effects and the mechanism by which MS affects cognitive functions in rodent models and what these cognitive changes are in different postnatal manipulations remain largely unknown.

In this study, two types of stress models (EH and PMS) were used to elucidate their effects on the behavior and endocrine function of offspring, as well as the relationship between BDNF expression in PFC and anxiety and spatial memory behavior in adolescent rats that had experienced early stress. We hypothesize that more severe forms of ELS (e.g. PMS) cause more severe suppression of BDNF compared to EH and control groups, and the corticosterone (CORT) levels may play a key role in this.

## Materials and Methods

### Animals

Maternal Sprague Dawley (SD) rats were purchased from the Laboratory Animal Center of Weifang Medical University (Weifang, China). Prior to the experiment, a simple field experiment was performed to screen the female rats for mating. The inactive, stressed or anxious rats were screened out by observing the extent of spontaneous activity to avoid influencing the experimental results. Animal experiments and handling were conducted in accordance with the National Institutes of Health guidelines (Use of Laboratory Animals) and were approved by the Weifang Medical University Animal Care and Use Committees. Upon arrival, the SD rats were allowed to acclimatize for 1 week, prior to impregnation, with food and water *ad libitum*. Maternal SD rats conceived pups in their natural state without external stimulation during pregnancy. The rats were housed in temperature-controlled (23 ± 2°C) and humidity-controlled (50 ± 5%) rooms, and maintained on a 12-h light/12-h dark cycle (lights on from 07:00 to 19:00) with food and water *ad libitum*. In order to minimize the potential confounding effect of litter (28), each experimental group consisted of rats derived from at least three different litters, and 3‑5 pups in each litter. Neonatal SD rats and their male offspring were used in this study. On PND 21, the male animals were weaned and housed in same-sex groups.

### Maternal Separation (MS) Procedure

Male offspring were randomly assigned to the EH group (MS for 15 min daily; 08:00‑08:15 a.m.; n=11 L), the PMS group (MS for 180 min daily; 08:00‑11:00 a.m.; n=11 L), and the control (CON) group (no separation; n=11 L) from PND 1‑21 (29, 30). During the separation process, the mother was transferred into a holding cage placed in an adjacent room to minimize olfactory and auditory communication with the pups. During this period, the pups from both EH and PMS conditions were removed from the home cage and kept in an incubator at 30 °C. At the end of the separation, the pups and the mothers were returned to their maternity cages. The control subjects were exposed to the normal husbandry procedures in the animal facility.

### Body Weight Gain

The body weight of the offspring was recorded weekly, during PND 7‑56, to ensure that the body weight was not a confounding factor and did not affect the behavioral results.

### Open Field Test

The OFT is typically performed to measure the locomotor activity and anxiety-like behaviors in rodents (31). The selected rats from each group were evaluated by the OFT during their dark cycle (between 01:00 and 05:00 p.m.) on PND 42. The apparatus used consisted of a square box (80 cm × 80 cm × 50 cm), with a field that was divided into 25 squares using the Smart-3.0 video tracking system (Panlab S.L., Barcelona, Spain). During the test, each rat was placed in the center of the open field and allowed to explore the arena freely for 5 min. The number of crossings (all four limbs), upright, carding, fecal grains, and time spent in the central area of the apparatus were observed. The apparatus was cleaned with 70% ethanol between each test to eliminate any olfactory cues left by the previously rat.

### Y-Maze Test

Cognitive and motor performances of the offspring of rats were assessed in a Y-maze, as it is strongly associated with the PFC (32, 33). The Y-maze apparatus consisted of three arms made of black opaque metal plates. Each arm was 50 cm long, 30 cm high, 18 cm wide, and at an angle of 120 degrees with the other two arms. Rats were habituated to the Y-maze for 3 days (between 08:00 a.m. and 11:00 a.m., 5 min explorations per day) from PND 42 onwards. Following habituation, the rats were trained to perform the task, where one branch arm was blocked to force the animal to enter the open branch arm. Then the animal received one food pellet (only one food was given for each training), and they were free to explore in the maze for 10 min (training trial). The mice were then returned to their cages. One hour later, the rats were reintroduced into the starting arms and allowed to freely explore all three arms for 5 min (test trial). The following parameters were measured: total number of arm entries in the maze and percent novel arm entry alternation (number of alternations/number of arm entries- 2).

### Enzyme-Linked Immunosorbent Assay (ELISA) Analysis

To determine the influence of MS on the blood CORT levels, rats were euthanized with 8% chloral hydrate (400 mg/kg, I.P.). Heart blood samples were collected in the morning (between 08:00 and 09:00 a.m., time of secretion peak) for the ELISA analysis on PND 57. The heart blood samples were drawn into EDTA-coated tubes, maintained on ice, centrifuged at 4,000 g for 10 min, and then the plasma was collected. The CORT concentration was measured using a rat corticosterone, CORT, ELISA kit (AD3193Ra; Andygene Biotechnology Co., Ltd., Beijing, China), according to the manufacturer’s protocol. The samples (or standard) and the conjugate were added to each well and the plate was incubated for 1 h at room temperature without blocking. The wells were washed several times with various buffers and the proper color developed. The plasma CORT levels were detected by absorbance of samples at 405 nm, which were compared to known CORT standards using an automated Epoch microplate spectrometer and the KinetiCalc Jr. Software (Bio-Tek Instruments, Winooski, VT, USA).

### Immunohistochemistry Analysis

The immunohistochemistry analysis was performed in accordance to a previous report (34). Three rats in each group were euthanized and transcardially perfused (0.01 M cold phosphate-buffered saline (PBS) for 5 min followed by 4% cold paraformaldehyde for 20 min). The brain of each rat was then stored at 4°C in 4% paraformaldehyde for the immunohistochemistry analysis. The PFC of the brain was transferred into 30% sucrose in 0.1 M cold PBS (4°C) until it sank. Coronal slices (20 µm thick) from the bregma (2.00 to 4.00 mm) were sectioned as the PFC and collected on the poly-lysine-coated slides. Each section was categorized anatomically according to the distance from the Bregman by using the rat brain atlas (35). The sections were dried for 2 h at 60°C and then treated with 0.3% H_2_O_2_ in methanol for 15 min to inactivate nonspecific peroxidase reactions. The sections were rinsed three times with PBS for 15 min and then the sections were blocked with 4% normal goat plasma at room temperature for 30 min. The primary antibody against BDNF (Anti-BDNF, 1:500, ab108319; Abcam, Cambridge, UK) was diluted with primary antibody dilution buffer and incubated at 4°C overnight. After repeated washing, the sections were incubated with the secondary antibody (Horseradish peroxidase conjugated affinity purified goat anti-rabbit IgG (H + L), 1:200 dilution, ZB-2301; ZSGB-Bio Co., Beijing, China) for 20 min at room temperature. The samples were rinsed and incubated for an additional 30 min to react the samples with a DAB (3,3’-diaminobenzidine) kit (ZLI-0931; ZSGB-Bio Co.) for color reactions at room temperature. The sections were counterstained with hematoxylin for 3 min, rinsed, dehydrated, cleared in xylene, and cover-slipped. The sections were subsequently examined on an Olympus FluoView 1200 confocal microscope system (Olympus Corp., Tokyo, Japan), and three images of representative PFC areas (400 × and 200 ×) were acquired per animal and the numbers obtained in each image were averaged. The number of positive cells was calculated using ImageJ software.

### Western Blot Analysis

**Figure 1:**
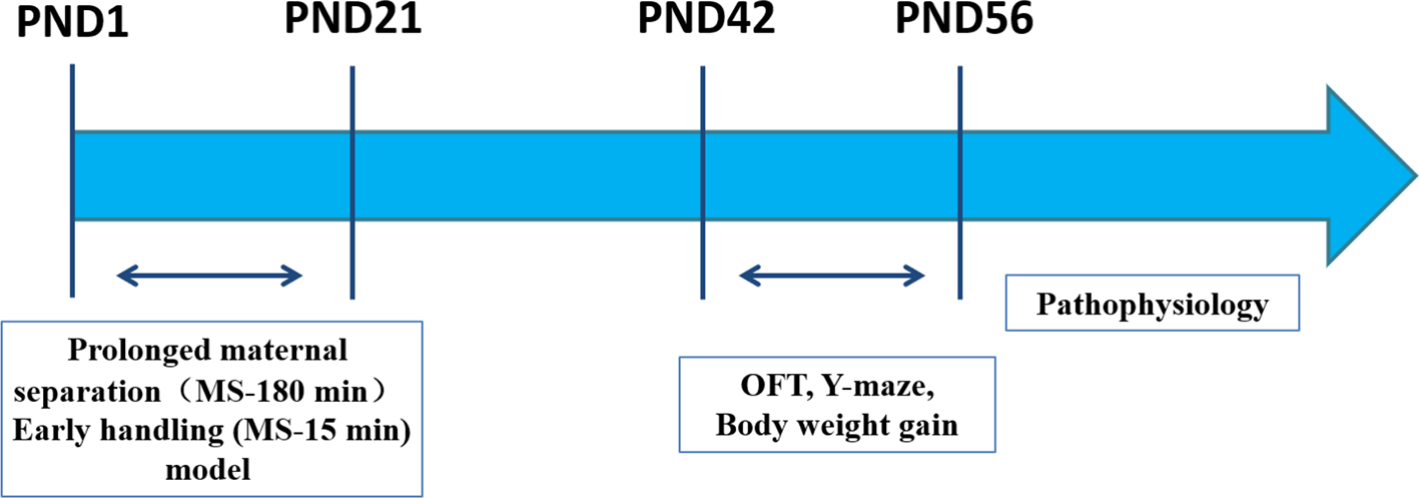
Experimental outline. Male offspring rats were studied in parallel throughout the experiment. Two postnatal manipulations were used. PMS (MS-180): Prolonged maternal separation (MS) consists of daily separation of offspring from their mother for 180 min PND 1‑21. EH (MS-15): Early handling daily 15-min separation of offspring from their mother on PND 1‑21. Pathophysiology (ELISA analysis, immunohistochemistry analysis, Western blot analysis). PND: postnatal day. OFT: Open-field Test on PND 42. Y-maze on PND 42‑44. Body weight gain on PND 7‑56.

The Western blot analysis was performed in accordance with a previous publication (36). The PFC was collected and homogenized on ice (n= 3). The protein of the PFC was extracted with a RIPA buffer (P0013B; Beyotime, Shanghai, China) containing the protease inhibitor cocktail (ST506, Beyotime) on ice. The bicinchoninic acid (BCA) assay was used to determine the protein concentration. Equal amounts of protein samples (30 μg) were denatured by heating at 95°C for 10 min, separated by sodium dodecyl sulfate-polyacrylamide gel electrophoresis (SDS-PAGE) using a 10% SDS-PAGE gel (ran for 1.5 h at 120 V). Then, separated proteins were transferred onto a polyvinylidene difluoride (PVDF) membrane using a Trans-Blot wet transfer system (transferred for 2 h at 110 mA) with a transfer buffer for Western blot analysis. After transfer to the membrane, the PVDF membrane was blocked with 5% non-fat dry milk, in 0.05% Tween-20 in PBS (PBST). Subsequently, the membrane was incubated overnight at 4°C with the primary antibody (anti-BDNF, 1:1000, ab108319, Abcam). The following day, the membranes were subjected to three sequential 5 min washes with PBST, washed three times for 10 min with PBST, and then incubated for 1 h at room temperature with the secondary antibodies (Horseradish peroxidase-conjugated affinity purified goat anti-rabbit IgG (H + L), 1:200 dilution, ZB-2301, ZSGB-Bio Co.). The protein bands were visualized in an electrochemiluminescence (ECL) solution (WBKLS0050; Millipore, Billerica, MA, USA) for 1 min and the gray value of the immunoreactive protein band was quantified with the Image J software (NIH, Bethesda, MD, USA). An overview of the experimental procedure is depicted in **Figure 1**.

### Statistical Analysis

All data were expressed as the mean ± standard error of mean (SEM) for the groups. The differences among groups in behavioral and biochemical results were analyzed with one-way analysis of variance (ANOVA) using the SPSS statistical software (Version 22.0, IBM Corp., Armonk, NY, USA), followed by a Fisher least significant difference (LSD) *post hoc* test. The body weight was analyzed by repeated-measures ANOVA. Pearson’s correlation coefficient r was calculated to determine the specific relationship between the BDNF levels in the PFC, plasma CORT concentration and the behaviors of rats, respectively. In addition, the Kolmogorov-Smirnov test and Levene’s test were used to evaluate the normality and variance homogeneity of the data distribution. The differences were considered statistically significant when p<0.05.

## Results

### Effects of Exposure to Different Postnatal Manipulations on the Body Weight of Pups

**Figure 2:**
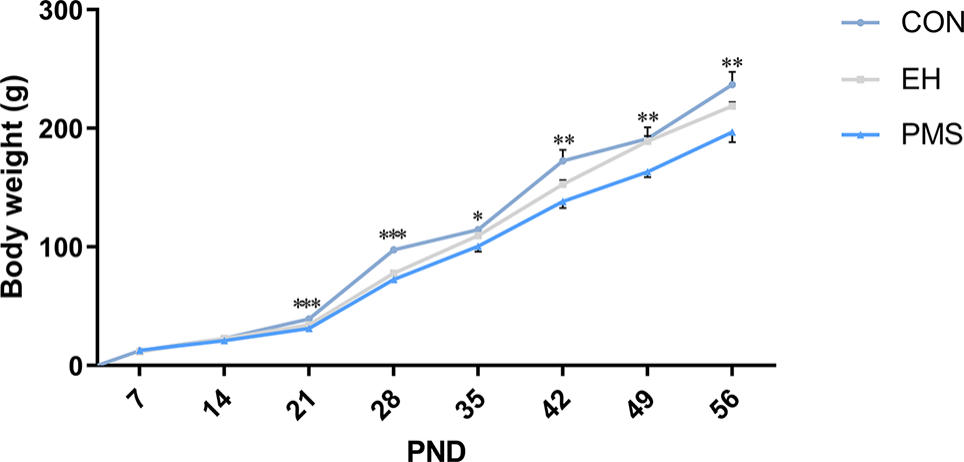
Body weight of offspring rats during experimental procedure. Data are presented as the mean body weight of the pup in each stage. Body weight differences in the control group and EH group and PMS group (CON, EH, PMS). Points represent mean ± SEM, (n=11 rats per group). ^*^p < 0.05, ^**^p < 0.01, ^***^p < 0.001.

The average body weight increase of the offspring of each group from PND 7‑56 is shown in **Figure 2**. During PND 7‑21, the mean pup weight of PMS and EH litters was similar. Furthermore, in several time points during PND 21‑56 [PND 21: F (2, 30)= 11.526, p= 0.000; PND 28: F(2, 30)= 53.096, p= 0.000; PND 35: F(2, 30)= 4.206, p= 0.025; PND 42: F(2, 30)= 6.926, p= 0.003; PND 49: F(2, 30)= 5.480, p= 0.009; PND 56: F(2, 30)= 5.887, p= 0.007], there were significant differences in body weight among the groups. ANOVA results showed that age [F(7, 24)= 831.588, p= 0.000] and PMS [F(14, 50)= 4.714, p= 0.000] had a significant effect on the body weight of rats, and there was an interaction between the two treatment factors. Further analysis showed that the pups in the PMS and EH groups grew more slowly than the pups in the CON group. Moreover, on PND 28 and PND 49, the weight of rats in the PMS group decreased significantly compared with that in the EH group (PND 28: p= 0.047; PND 49: p= 0.010).

### Effects of Exposure to Different Postnatal Manipulations on the Spontaneous Activity of Pups

**Figure 3:**
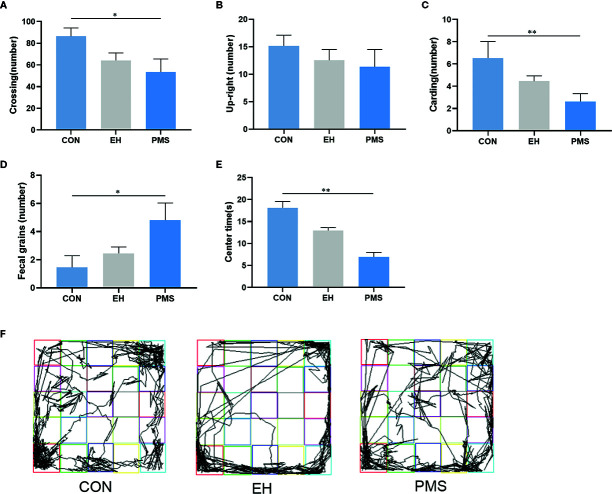
In the open field, PMS group had reduced number in upright, carding and fecal grains compared with other groups**. (A)**: the number of crossings in the OFT. **(B):** the number of upright in the OFT. **(C):** the number of carding in the OFT. **(D):** the number of fecal gains in the OFT. **(E):** the time at the center state in the OFT. Values are expressed as the mean ± SEM (n= 11 rats per group). Representative diagrams of the pathways in the OFT from each group, and **(F)** showed the rats’ mobile trajectory Figs in CON, EH and PMS group. ^*^p < 0.05, ^**^p < 0.01.

The OFT results revealed significant differences in the number of crossings [F(2, 30)= 3.425, p= 0.046, **Figure 3A**], carding [F(2, 30)= 3.922, p= 0.031, **Figure 3C**], fecal grains [F(2, 30)= 3.771, p= 0.035, **Figure 3D**] and the time spent in the central area [F(2, 30)= 18.613, p= 0.000, **Figure 3E**] among the three groups of rats, but there was no significant difference in the number of upright [F(2, 30)= 0.653, p= 0.528, **Figure 3B**]. Further data analysis revealed that reduction of the number of crossings (p= 0.016), carding (p= 0.009), and fecal grains (p= 0.012) was observed in the PMS group of rats compared to control rats. Additionally, the time in the central area of the rats was also significantly reduced (p= 0.000), but there was no significant difference between the EH group and the CON group. The tracking images were shown in the figure (**Figure 3F**).

### Effects of Different Postnatal Manipulations on the Cognitive Behavior of Pups

**Figure 4:**
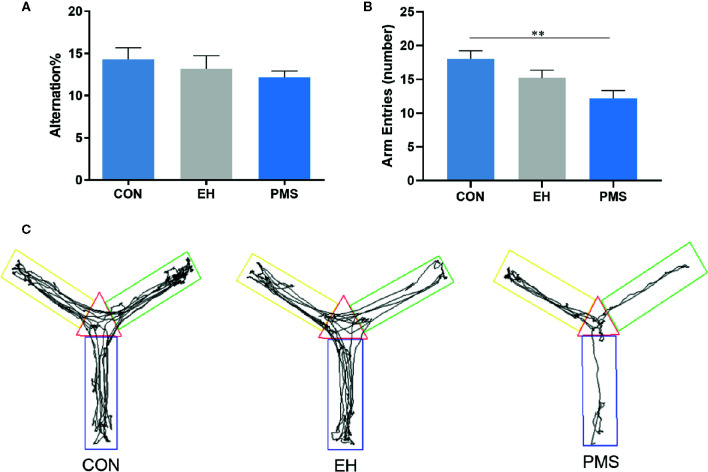
Comparative effects of two different separation modes in the PMS and EH group on spontaneous Y-maze alternation performance of rats [**(A)**: alternation %, **(B)**: number of arm entries]. Each bar represents the mean ± SEM (n= 11 rats per group). Representative diagrams of the pathways in the Y-maze test from each group, **(C)** showed the rats’ mobile trajectory Figs in CON, EH and PMS group. ^**^p < 0.01.

As shown in **Figure 4**, the motor performance values (arm entries) showed significant differences among the groups [F(2, 30)= 6.134, p= 0.006], although the cognitive performances values (alternation %) did not show significant differences [F(2, 30)= 0.716, p= 0.497]. The number of arm entries in the PMS group was significantly lower than that in the control rats (p= 0.001). Exposure to EH (EH group) had no significant effect on the measured parameters. The EH and the PMS groups did not have any significant effects on the alternation %. The distance traveled indicated the differences in the space exploration between the two separation models (EH and PMS) and the control rats (**Figure 4C**).

### Effects of Exposure to Different Postnatal Manipulations on the CORT Levels in the Plasma of Pups

**Figure 5:**
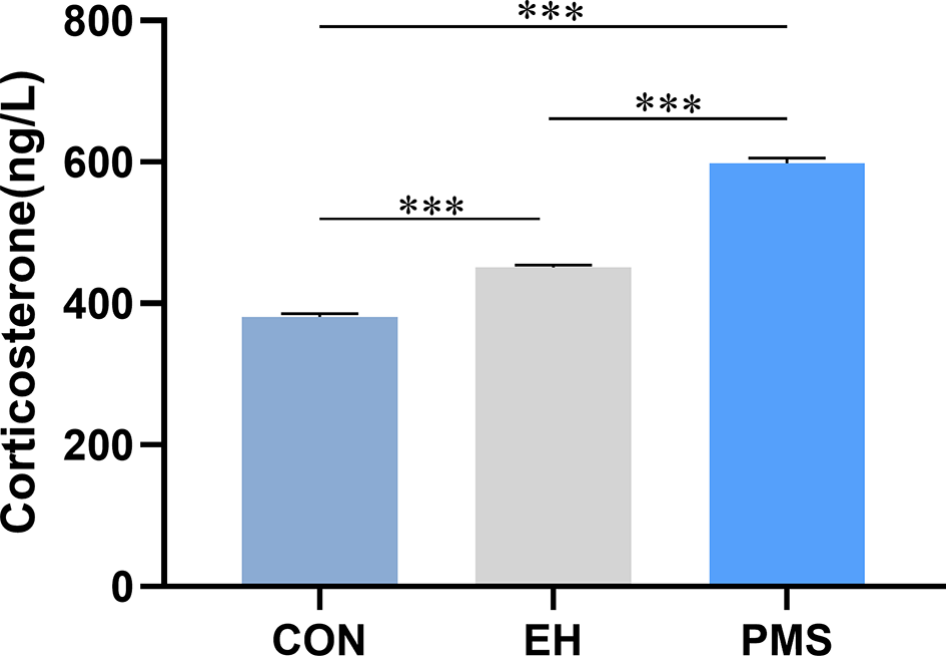
Corticosterone (CORT) in puberty. Levels of circulating plasma CORT were assessed in the control group, EH group and PMS group (CON, EH, PMS) on PND 56. Values are expressed as the mean ± SEM (n= 11 rats per group). ^***^p < 0.001.

The effects of exposure to EH (EH group) and PMS (PMS group) on the plasma CORT levels were determined as shown in **Figure 5**. There were significant differences in the CORT expression levels among the three groups [F(2, 30)= 439.177, p= 0.000]. Compared with the levels of CORT in the control group, the CORT contents were significantly increased in the PMS group (p= 0.000) and the EH group (p= 0.000).

### Effects of Exposure to Different Postnatal Manipulations on the Expression Levels of BDNF Protein in the PFC of Pups

#### Immunohistochemistry Analysis

**Figure 6:**
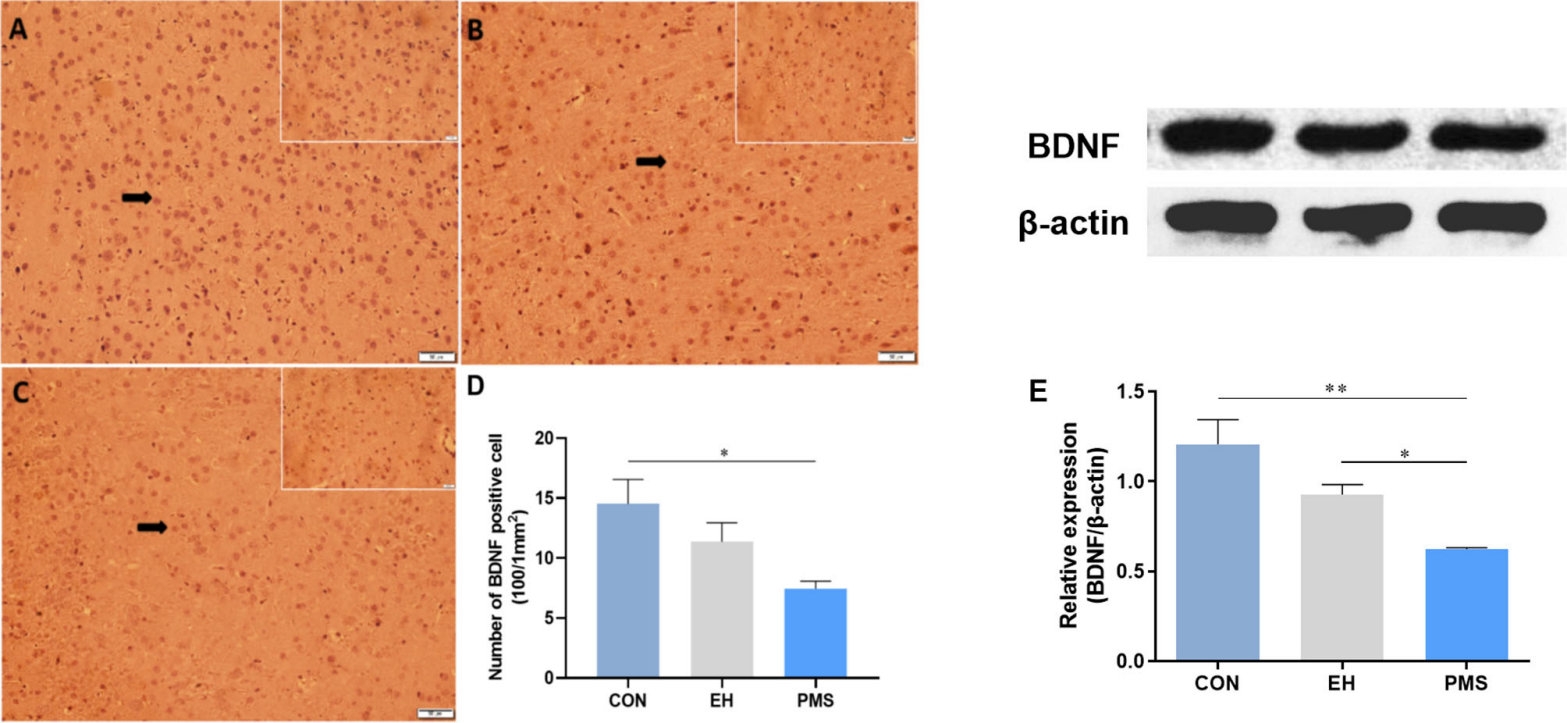
Effects of the PMS and EH procedures on the levels of BDNF protein in the PFC. **(A)**: The figure displays BDNF immunostaining in the PFC from the CON group. **(B)**: A section of the PFC of the EH group showed the presence of BDNF positively stained cells in the outer layer of the PFC. **(C)**: The section from rats of the PMS group showed more BDNF positively stained cells in the outer layer of the PFC. Magnification 200× for **(A), (B),** and **(C)**, and 400× for the inserts (arrow). **(D)**: Quantification of the difference in BDNF positive cells in the PFC between groups. **(E)**: The Western blot analysis results of BDNF in the PFC samples from the EH, PMS and control (CON) groups. A sample of 30 μg of cellular protein from different rat brain regions was processed for Western blot assays using antibodies against BDNF and β-actin. The Intensity of the immunoreactive protein was measured using the Image J software. Values are expressed as the mean ± SEM (n= 3 rats per group). *p < 0.05, **p < 0.01.

The number of BDNF-positive cells in the CON, EH, and PMS groups is shown in **Figure 6**. The expression of BDNF positive cells showed significant differences among the three groups [F(2, 6)= 5.362, p= 0.046] (**Figures 6A–C**). Compared with the CON group, the estimated total number of BDNF-positive cells in the PFC was significantly lower (p= 0.017) in the PMS group (**Figure 6D**), but there was no significant effect on the EH group within the mean number of the BDNF-positive cells.

#### Western Blot Assays

The BDNF protein levels were determined by measuring the protein bands expressed in the PFC (**Figure 6E**). The BDNF protein expression levels showed significant differences among the three groups [F(2, 6)= 10.134, p= 0.012]. Further analysis by LSD showed that the optical density of BDNF in the PFC of the PMS group was significantly lower than that in the CON group (p= 0.004). Although the average density of the BDNF protein in the EH group was also decreased, there was no significant difference between the EH group and CON group (p= 0.037).

### Correlation Between BDNF Levels in the PFC, Plasma CORT Concentration and Behavioral Activity in Rats

**Figure 7:**
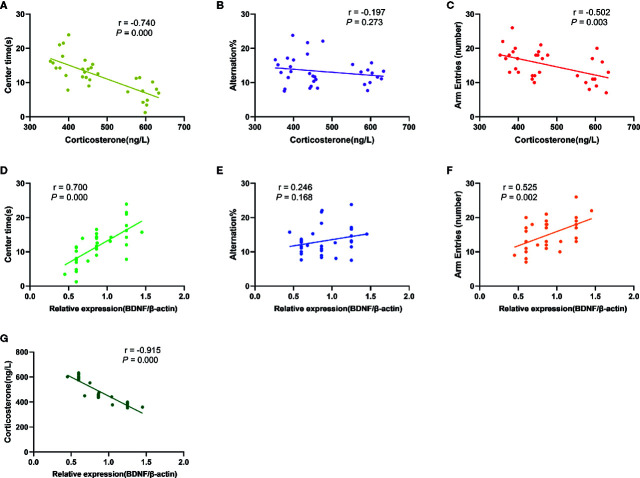
Results of correlation analysis. **(A)**: The correlation between plasma CORT levels and time spent in the central area of rats in OFT (r= -0.740, p= 0.000); **(B)**: The correlation between plasma CORT levels and alternation % in Y-maze test (r= -0.197, P= 0.273); **(C)**: The correlation between plasma CORT levels and number of arm entries of rats in Y-maze test (r= -0.502, p= 0.003); **(D)**: The correlation between BDNF protein expression in the PFC and time spent in the central area of rats in OFT (r= 0.700, p= 0.000); **(E)**: The correlation between BDNF protein expression in the PFC and alternation % in Y-maze test (r= 0.246, p= 0.168); **(F)**: The correlation between BDNF protein expression in the PFC and number of arm entries of rats in Y-maze test (r= 0.525, p= 0.002); **(G):** The correlation between BDNF protein expression in the PFC and plasma CORT levels (r= -0.915, P= 0.000).

The results of correlation analysis (**Figure 7**) showed that the plasma CORT levels were negatively correlated with time spent in the central area (OFT; r= -0.740, p= 0.000) and number of arm entries (Y-maze; r= -0.502, p= 0.003) of rats respectively, but not with the alternation % (Y-maze; r = -0.197, P = 0.273). In contrary, BDNF levels in the PFC exhibited positive correlation with time spent in the central area (r= 0.700, p= 0.000) and number of arm entries (r= 0.525, p= 0.002), but no correlation with alternation % (r= 0.246, p= 0.168). There was also a significant negative correlation between plasma CORT concentration and BDNF levels in the PFC (r = -0.915, P = 0.000).

## Discussion

The aim of the present study was to examine the consequences of two models of ELS that differ in terms of their severity on anxiety-like behavior and spatial memory as well as the BDNF levels in the PFC and plasma CORT levels. The results indicated the variations in postnatal separation not only influenced the development of spontaneous activity and endocrine responses to stress in the offspring but also decreased the expression levels of BDNF in the PFC.

The results of the present study found a significant increase in anxiety-like behaviors in rats exposed to PMS but not in offspring exposed to EH. This finding was consistent with a recent meta-analysis showing increased anxiety in rats exposed to PMS, that was more pronounced in younger animals (37). Further, these outcomes supported the notion that more severe forms of stress early in life increased the risk of several behavioral abnormalities, including anxiety, later in life. The lack in anxiety-like behavior in offspring exposed to EH was similar to findings reported by Eklund (38). However, others have shown that brief postpartum manipulations attenuates fear and anxiety responses (39, 40). This discrepancy was likely due to differences in methodology related to the handling procedure and the sensitivity of anxiety-like behavior to external cues such as lighting conditions, olfactory cues, time of testing, and sex of the tester (41–44).

Notably, these anxiety-related mood changes may affect the growth and development of the pups and their weight gain (45, 46). Contradictory findings have been reported about the effect that exposure to MS has on body weight, certain studies found that the body weight in pups was lower in adulthood, while other studies found no differences between stressed and control animals (47). Although the reasons behind these discrepancies remain unclear, the differences in stimulation intensity and neuroregulatory function could play significant roles in the outcomes (48). The present study found no significant difference in the average weight of pups among the three groups at PND 7 and PND 21. This finding might indicate that the food intake and weight gain of the offspring are not affected during the MS procedure as long as the pups are returned to feeding after separation. During the PND 21‑56 period, the body weight of the PMS group animals was significantly different from that of the CON group rats, but there was no significant difference between the EH group rats and the CON group rats. Additionally, this study found that EH rats had lower body weight during the PND 21‑28 period, although the body weight difference between the EH and CON groups disappeared in adolescence. These results indicated that PMS caused a more significant reduction in body weight compared to pups exposed to EH and was consistent with clinical findings showing that severe forms of adversity lead to stunted growth (49). A possible mechanism for the effects of PMS on weight and anxiety might be due to higher basal levels of CORT found in PMS animals. This assertion was supported by our finding that CORT levels were highly correlated with anxiety-like behavior (**Figure 7A**).

Studies using experimental models of early stress have also shown that the imbalance of stress hormones can lead to cognitive impairment (50). Adolescence is a period of extensive brain maturation in which synaptic balance and stability are developed and cognitive function and synaptic plasticity are matured (51). PFC is one of the key brain areas involved in cognitive and emotional function regulation. The synaptic plasticity of this region is very important for learning and memory processes and highly regulated by early environmental factors. Moreover, disturbances in the maturation of the PFC have been linked to the emergence of multiple mental illnesses during adolescence (52). It has been reported that ELS can interfere with the maturation process of PFC, damage the structure and function of prefrontal neurons, which is manifested in the decreased number of neurons and synaptic transmission efficiency (53). However, little is currently known about the effects of EH and PMS on PFC development and function. Although, EH and PMS did not affect alternation % in the Y-maze, there was a significant reduction in BDNF levels in the PFC of PMS rats compared to EH and CON (**Figure 6**). This was important because BDNF mediates long-term potentiation (a form of synaptic plasticity) in the PFC and was considered as the key biological mechanism underlying associative learning and memory recognition (54). Genetic analysis revealed that Val66Met, the BDNF allele, was associated with a high risk of suicide ideation, emotional vulnerability to stressful life (55), and prefrontal cortex atrophy (56). Similar behaviors were found in BDNF +/met knock-in mice that exhibited depression- and anxiety-like behaviors, and impaired working memory after stress exposure (57). This polymorphism seemed to also predict the stress response and remission incidence (58). In addition, high concentration CORT have been shown to inhibit the secretion of BDNF in the hippocampus (59). This finding was consistent with our findings that CORT levels were negatively correlated with BDNF expression in the PFC (**Figure 7G**). Interestingly, BDNF levels were positively correlated with the time spent in the center of the open field (**Figure 7D**) raising the possibility that levels of BDNF in the PFC may play a role in programming anxiety-like behavior in juvenile male rats. Additional studies are needed to clarify whether similar changes in BDNF levels in the PFC and anxiety are also seen in female rats exposed to PMS.

Despite the large variation in ELS models, species, test age, and outcome parameters, most of the studies only reported slight behavior changes in females after experiencing early life adversity (60). According to the literature, ELS only affected the survival levels of adult-born neurons in males associated with a more seriously damaged cognitive performance in adult ELS-male rodents compared with females (61). However, we found that male rats with early stress, as revealed in the Y-maze test, showed no significant change in spatial cognitive behavior, even if the alternation rate decreased, which may only indicate the risk of potential cognitive impairment, thus requiring more cognitive behavioral tasks to advance future research on rodents of different ages and genders. In addition, it has been found that the behavior and neural outcomes of individuals exposed to ELS have changed, and that these outcomes can be passed on to their offspring (62). Therefore, it will be worthwhile to explore whether female rats exposed to ELS can transmit stressed phenotypes across generations as well as its potential epigenetic mechanisms.

## Conclusion

In summary, this study demonstrated that PMS increased CORT secretion, down-regulated BDNF expression in the PFC, and induced anxiety-like behavior in adolescent pups. Exposure to brief separation (EH) did not alter anxiety-like behavior, or BDNF levels, but increased CORT levels compared to CON, but to significantly lower levels compared to PMS. These findings are consistent with a large body of clinical work suggesting that more severe forms of ELS cause more significant behavioral and physiological abnormalities. Further, our findings suggest a possible role for elevated CORT in programming BDNF levels and abnormal PFC function in more severe forms of ELS.

## Data Availability Statement

The original contributions presented in the study are included in the article/supplementary material; further inquiries can be directed to the corresponding author.

## Ethics Statement

The animal study was reviewed and approved by Weifang Medical University Animal Care and Use Committees.

## Author Contributions

LS, XZ, and HL are responsible for the conception and design of the work. HaS, YJ, XS, GZ, and ZD are responsible for the acquisition and analysis of data for the work. QL is responsible for drafting the work for important content. HoS and YK participated in the revision of the article. All authors contributed to the article and approved the submitted version.

## Funding 

This work was supported by the Medicine and Health Science and Technology Development Project of Shandong Province (2018WS213), the Youth Natural Science Foundation of Shandong Province (ZR2017QH001), the Science and Technology Foundation Program of Colleges and Universities of Shandong Province (J17KB096), the Students’ Research Fund of Weifang Medical University (KX2019062).

## Conflict of Interest

The authors declare that the research was conducted in the absence of any commercial or financial relationships that could be construed as a potential conflict of interest.
